# Regular-Fat Dairy and Human Health: A Synopsis of Symposia Presented in Europe and North America (2014–2015)

**DOI:** 10.3390/nu8080463

**Published:** 2016-07-29

**Authors:** Arne Astrup, Beth H. Rice Bradley, J. Thomas Brenna, Bernadette Delplanque, Monique Ferry, Moises Torres-Gonzalez

**Affiliations:** 1 Department of Nutrition, Exercise and Sports, University of Copenhagen, Frederiksberg C DK-1958, Denmark; ast@nexs.ku.dk; 2 Foodsense, LLC, 167 Lyman Ave., Burlington, VT 05401, USA; 3 Division of Nutritional Sciences, Cornell University, Savage Hall, Ithaca, NY 14853, USA; jtb4@cornell.edu; 4 Institut des Neurosciences Paris-Saclay (Neuro-PSI), Universite Paris-Sud, Bat 447, Orsay 91405, France; Bernadette.delplanque@u-psud.fr; 5 Sorbonne Paris-Cité, 190 Avenue de France, Paris 75013, France; monique.ferry@club-internet.fr; 6 National Dairy Council, 10255 West Higgins Road, Suite 900, Rosemont, IL 60018, USA; Moises.Torres-Gonzalez@Dairy.org

**Keywords:** dairy, fat, milk, cheese, yogurt, cardiovascular disease, Type-2 diabetes, infant formula

## Abstract

In recent history, some dietary recommendations have treated dairy fat as an unnecessary source of calories and saturated fat in the human diet. These assumptions, however, have recently been brought into question by current research on regular fat dairy products and human health. In an effort to disseminate, explore and discuss the state of the science on the relationship between regular fat dairy products and health, symposia were programmed by dairy industry organizations in Europe and North America at The Eurofed Lipids Congress (2014) in France, The Dairy Nutrition Annual Symposium (2014) in Canada, The American Society for Nutrition Annual Meeting held in conjunction with Experimental Biology (2015) in the United States, and The Federation of European Nutrition Societies (2015) in Germany. This synopsis of these symposia describes the complexity of dairy fat and the effects regular-fat dairy foods have on human health. The emerging scientific evidence indicates that the consumption of regular fat dairy foods is not associated with an increased risk of cardiovascular disease and inversely associated with weight gain and the risk of obesity. Dairy foods, including regular-fat milk, cheese and yogurt, can be important components of an overall healthy dietary pattern. Systematic examination of the effects of dietary patterns that include regular-fat milk, cheese and yogurt on human health is warranted.

## 1. Introduction

Summary of lecture presented by Dr. Mario Kratz at The American Society for Nutrition Annual Meeting held in conjunction with Experimental Biology, 2015 (USA) [1]. The following text represents the authors’ summary of Dr. Kratz’s lecture. Dr. Kratz was not involved with the writing or publication of the text.

Over the last three decades, dietary guidelines have concluded that dairy fat is an unnecessary source of calories and saturated fat in the human diet [2,3]. The assumption that consuming foods containing saturated fat, such as dairy foods, increases the risk for cardiovascular disease has led nutrition authorities to advise the consumption of low-fat and fat-free dairy foods instead of regular-fat dairy foods [3,4]. Further, there is a belief in the scientific community that by removing fat from dairy products, caloric intake may be reduced [2].

These assumptions, however, have recently been questioned by current research on regular fat dairy products and human health. The first assumption, that dairy foods are an unnecessary source of saturated fat, does not take into account that dairy fat contains nearly 400 different fatty acids, of which saturated fatty acids are a part, constituting the largest proportion of total dairy fat [5]. Dairy fat is the most complex fat source in the human diet, and contains a number of unique fatty acids such as short- and medium-chain fatty acids, *trans*-fatty acids and branched-chain fatty acids, all of which have biological significance [6]. The second assumption, that a diet rich in dairy fat will raise plasma low-density lipoprotein (LDL)-cholesterol and therefore risk of cardiovascular disease, does not take into account the biological differences between individual saturated fatty acids, the complexity of the dairy food matrix, and the multi-factorial nature of heart disease, whereas the replacement of unsaturated fatty acids with saturated fatty acids, such as C16:0 and C18:0, the most abundant saturated fatty acids in milk fat, will indeed raise plasma LDL-cholesterol, it will also raise plasma high-density lipoprotein (HDL)-cholesterol [7]. Plasma HDL-cholesterol is associated with a decreased risk of cardiovascular disease. In addition, the increase in LDL-cholesterol will be in large buoyant LDL, which is more resistant to oxidation and therefore less atherogenic than small dense LDL [7]. Further, cardiovascular disease is a complex disease of which serum lipids are just one of several risk factors [8]. The third assumption, that the consumption of dairy fat will contribute to obesity and therefore cardiometabolic diseases, does not take into account the effect that regular-fat dairy foods have on satiety, glucose metabolism and several risk factors associated with cardiovascular disease and type 2 diabetes [9].

In an effort to disseminate, explore and discuss the state of the science on the relationship between regular fat dairy products and human health, symposia were programmed by dairy industry organizations in Europe and North America at The Eurofed Lipids Congress (2014) in France, The Dairy Nutrition Annual Symposium (2014) in Canada, The American Society for Nutrition Annual Meeting held in conjunction with Experimental Biology (2015) in the United States, and The Federation of European Nutrition Societies (2015) in Germany. This article is a synopsis of what was presented at those meetings regarding regular-fat dairy products and human health.

## 2. Dairy Fat: A Complex Mixture of Bioactive Fatty Acids

Summary of lecture presented by Dr. J. Thomas Brenna at The American Society for Nutrition Annual Meeting held in conjunction with Experimental Biology, 2015 (USA) [10].

Current dietary recommendations indicate there is strong evidence that replacing saturated fat with unsaturated fats, particularly polyunsaturated fat, reduces plasma LDL-cholesterol and cardiovascular disease risk [4]. According to the 2015 report of the Dietary Guidelines Advisory Committee (DGAC), strong and consistent evidence indicates that replacing saturated fatty acids (SFA) with polyunsaturated fatty acids (PUFA) reduces the risk of cardiovascular disease (CVD) events and coronary mortality [2]. This is the case, however, only if saturated fat is replaced by a mixture of *n*-6 and *n*-3 PUFA, because replacement with pure *n*-6 polyunsaturated fat may increase risk [11]. There is limited evidence that replacing saturated fat with monounsaturated fat reduces the risk of cardiovascular disease [2]. The report also indicates there is strong evidence that replacing saturated fat with carbohydrates does not lower risk of cardiovascular disease [2].

Humans eat complex foods containing a mixture of fatty acids, and thus dietary recommendations can be confusing to consumers, particularly when it comes to dairy foods (milk, cheese and yogurt). The major source of calories from cow’s milk is from saturated fat, but cow’s milk also contains monounsaturated fatty acids (MUFA) and PUFA, naturally occurring *trans*-fatty acids, as well as branched-chain fatty acids [5]. Further, the saturated fat in cow’s milk is made up of short-, medium- and long-chain fatty acids ranging from four to 18 carbons in length [5]. Therefore, to classify dairy fat as harmful saturated fat may be overly simplistic and misleading.

The numerous fatty acids in dairy fat are often grouped by categories of chemical classification, with SFA being the primary component of dairy fat [5]. Palmitic acid (C16:0) is the most abundant SFA in retail cow’s milk, about 30% of total SFA followed by stearic acid (C18:0) and myristic acid (C14:0), about 11% and 10% of total SFA, respectively, and then by short- and medium-chain SFA (C4:0–C12:0), which make up about 13% of the total SFA in dairy fat [5].

Saturated fat is known to raise total cholesterol, including HDL-cholesterol and LDL-cholesterol. Higher HDL-cholesterol is thought to reduce cardiovascular disease risk by reverse cholesterol transport, the inhibition of LDL-cholesterol oxidation, and thus prevention of subsequent inflammatory pathways [6].

Dairy fat also contains several *trans*-fatty acids [5]. At approximately 3% of total fatty acids in dairy fat, *trans*-11 18:1 (vaccenic acid) is the most abundant *trans*-fatty acid in retail milk [5]. Unlike the consumption of industrially produced *trans*-fatty acids, such as those present in partially hydrogenated oils, the consumption of naturally-occurring *trans*-fatty acids present in ruminant milk have not been associated with increased risk of cardiovascular disease when consumed in typical amounts [12,13]. Conjugated linoleic acids (CLA) are a class of unsaturated fatty acids contained in dairy fat that have double bonds adjacent to one another. The major conjugated linoleic acid in dairy fat is rumenic acid (*cis*-9, *trans*-11 18:2), a fatty acid that has been associated with antiatherogenic and anticarcinogenic effects in vitro and in vivo [14,15], though results in humans have been inconsistent. Evidence from randomized controlled trials indicates that dietary CLA does not affect plasma cholesterol [16,17]. A clinical trial indicated that whereas extremely high levels of vaccenic acid (10× times the amount typically consumed) had similar effects as industrially produced *trans*-fatty acids on risk factors for cardiovascular disease in healthy adults, high levels of *cis*-9, *trans*-11 18:2 (10–18× the amount typically consumed) lowered triacylglycerol and had no effect on other lipoprotein risk factors [18]. Similarly, another clinical trial in men found that, whereas high dietary intake of *trans*-fatty acids from any source, industrially- or ruminally-produced, including vaccenic acid, adversely affected plasma LDL and HDL cholesterol, intake of ruminally-produced *trans*-fatty acids, such as vaccenic acid, in moderate amounts that exceed current normal intake had neutral effects on plasma lipids and other cardiovascular disease risk factors [19]. Results from a recent randomized controlled trial in women, however, indicated that butter enriched with vaccenic acid lowered HDL cholesterol in overweight women despite having no effect on total plasma cholesterol, LDL cholesterol, apolipoprotein B, A-1, and triglyceride (TAG) concentrations when compared with a control diet [20].

Another class of fatty acids present in dairy fat, the bioactivity of which has not been thoroughly investigated, is branched-chain fatty acids. Branched-chain fatty acids are primarily saturated fatty acids with one or more methyl groups. They make up about 2% of the total fatty acids in cow’s milk [21]. The most abundant branched-chain fatty acids in cow’s milk are C17:0, anteiso and C15:0, anteiso. These two fatty acids make up approximately 30% and 28%, respectively, of total branched-chain fatty acids and nearly half a percent of total dairy fat, in retail cow’s milk [21].

Branched-chain fatty acids increase membrane fluidity, and are resistant to oxidation [22]. In fact, many species of bacteria utilize branched-chain fatty acids in their membranes, and evidence indicates that branched-chain fatty acids may help shape the human microbiome. For example, branched-chain fatty acids are present in vernix, the waxy coating that is unique to human fetuses and infants. When vernix is sloughed off during the third trimester of pregnancy, it is swallowed by the fetus, with approximately 90% of it being absorbed [23]. This makes vernix the first solid meal consumed by the growing fetus, consisting of approximately 30% (*w/w*) branched-chain fatty acids [23]. The branched-chain fatty acids absorbed through the consumption of vernix in amniotic fluid provide a normal term newborn with branched-chain fatty acids throughout the gastrointestinal tract. The branched-chain fatty acid profile of vernix differs from that of meconium, newborn excrement, indicating that branched-chain fatty acids consumed by human fetuses are not metabolically inert or used only as fuel. The newborn lumen contains branched-chain fatty acids, indicating that the branched-chain fatty acids provided by vernix are likely to support colonization of specific organisms of the gut microbiome [23,24]. The absence of vernix, as is observed in premature newborns, is associated with necrotizing enterocolitis and a dramatic change in the make up of the premature newborn’s microbiota [24].

Cow’s milk products, such as milk, cheese and yogurt, are major sources of fat in the American diet, with the intake of ruminant-derived branched-chain fatty acids being approximately 220 mg/day from dairy foods [21]. This is a substantial fraction of daily total fat intake, particularly when compared with other bioactive fatty acids, such as eicosapentaenoic acid (EPA) and docosahexaenoic acid (DHA) [21]. The role of branched-chain fatty acids in the composition of the gut microbiome, and the amount consumed as a percentage of total daily fat therefore warrants further study into this bioactive class of fatty acids present in regular-fat dairy foods.

This brief survey of the bioactive fatty acids present in dairy fat highlights the complexity of the role of regular fat dairy in human health. To understand human disease risk, however, it is prudent to examine the effect from actual foods consumed, such as regular-fat dairy foods, including milk, cheese and yogurt, rather than individual fatty acids, on human health outcomes.

## 3. Saturated Fat, Dairy Fat and Plasma Lipid Biomarkers for Cardiovascular Disease: Clinical Evidence

Summary of lecture presented by Moises Torres-Gonzalez at The American Society for Nutrition Annual Meeting held in conjunction with Experimental Biology, 2015 (USA) [25].

Since the discovery of correlations between dietary fat, serum cholesterol and cardiovascular disease, scientists have been discouraging the consumption of nutrient-dense foods that contain saturated fat and/or cholesterol, such as regular-fat milk, cheese and yogurt [26]. The associations that indicated LDL-cholesterol was associated with an increased risk of heart disease, whereas HDL-cholesterol was associated with a decreased risk of heart disease, led to the over simplification that intake of saturated fat is causally related to increased cardiovascular disease [27]. There are multiple examples from the literature, however, that LDL-cholesterol is just one mediator of the atherosclerotic and thrombotic processes, and beneficial effects of a diet on cardiovascular disease risk is not necessarily reflected in measurements of LDL-cholesterol [28]. Current evidence shows that cardiovascular events occur in patients with normal LDL cholesterol levels, and [29] some patients who have experienced a clinical event have normal, or in some instances high, levels of HDL cholesterol [30].

Additionally, despite clinical practice guidelines based on targeting a reduction in plasma LDL-cholesterol, still considered the main biomarker for CVD risk reduction [31], advances in the understanding of LDL metabolism indicate that LDL particles are heterogeneous, differing in size, density, and atherogenecity, with small dense LDL particles being more atherogenic than large LDL [32,33]. For instance, different studies have shown that patients with lipoprotein profiles characterized predominantly by smaller, denser LDL particles were at a higher risk for CVD than those with predominantly larger LDL [34,35,36,37]. The higher atherogenecity of smaller, denser LDL has been attributed to its lower affinity for LDL receptors, greater binding to arterial proteoglycans and higher oxidative susceptibility than more buoyant LDL [38]. Higher prevalence of smaller dense LDL has been also reported in metabolic diseases associated with increased risk of CVD, such as obesity, type 2 diabetes and metabolic syndrome [32,39,40,41]. Thus, this emerging evidence suggests that oversimplifying CVD risk by looking only at LDL-cholesterol levels could misinterpret and perhaps even underestimate CVD risk.

Further, the functionality of HDL-cholesterol affects its cholesterol efflux capacity, highlighting that concentration alone is not the best indicator of risk [42]. Raised plasma TAG are considered another independent risk factor for cardiovascular disease [43]. Higher plasma TAG are associated with increased small-dense LDL particles and decreased HDL-cholesterol [43]. Plasma TAG are affected by diet. Paradoxically, high fat diets lower TAG and high carbohydrate diets raise them [44]. Clinical evidence has shown that dietary carbohydrate as well as excess calories can increase hepatic TAG stores that after lipolysis and remodeling, ultimately result in increased small-dense LDL [45].

Another important aspect of cardiovascular disease is a presence of low-grade chronic inflammation [46]. Inflammatory factors such as C-reactive protein, tumor necrosis factor-α and interleukin-6 have all been associated with an increased risk of cardiovascular disease [46]. Therefore, to assess cardiovascular disease risk based on LDL- and HDL-cholesterol measurements alone is overly simplistic. However, this is what is often reported in the literature of nutrition studies. Results from one nested case-control study indicated that in addition to screening lipid levels, measurement of C-reactive protein could improve the identification of persons at risk for cardiovascular events [47].

### 3.1. Saturated Fat and Plasma Biomarkers for Cardiovascular Disease: Beyond LDL-Cholesterol Levels

Current dietary recommendations for saturated fat are based on targeting a reduction in plasma LDL-cholesterol, with the consumption of saturated fat recommended to be no more than 7%–10% of calories [2,48,49]. A systematic review and meta-analysis of randomized trials that tested the effects of increased consumption of polyunsaturated fats in place of saturated fat on clinical endpoint of cardiovascular disease found that over time, replacing saturated fat with polyunsaturated fats significantly reduced the risk of coronary heart disease (CHD) [50]. On the other hand, recent publication of recovered clinical data from the 1970s [51] refuted the hypothesis that lowering serum cholesterol invariably translates into lowered risk of death from all-causes or CHD. The Minnesota Coronary Experiment (MCE) was a National Institutes of Health (NIH) supported double blind randomized control trial (RCT) led by Frantz and Keys to test whether the replacement of saturated fat with linoleic acid rich corn oil reduced CHD and death by lowering serum cholesterol. While a high corn oil diet significantly lowered serum cholesterol in accord with the Keys equation, it did not produce a benefit for mortality risk or risk of atherosclerosis or myocardial infarcts [51]. In fact, a 22% increased risk of death was observed for each (0.78 mmol/L) reduction in serum cholesterol, bringing into question the effectiveness of cholesterol reduction as a means to reduce the risk of death. Further, when included in a systematic review and meta-analysis of RCTs that lowered serum cholesterol by providing linoleic acid rich vegetable oil in place of saturated fat, no benefit to mortality or CHD risk was detected [51]. The results of this RCT and meta-analysis are inconsistent with the hypothesis that replacing saturated fat with a diet high in linoleic acid lowers all-cause mortality or the risk of death from CHD, despite lowering of serum cholesterol [51]. Analysis of emerging evidence suggests that the hypothetical benefit of replacing saturated fat with linoleic acid rich vegetable oil may have been overestimated, and warrants further investigation. Dietary changes that reduce cardiovascular events can take place without any effect on LDL- or HDL-cholesterol values, indicating that other mediators may be more important [52].

Evidence from large randomized clinical trials shows that reducing the percentage of calories from saturated fat to less than or equal to 10% of total calories does not necessarily improve plasma biomarkers for cardiovascular disease, because these biomarkers do not account for the complexity of cholesterol metabolism [53]. For example, the Dietary Approaches to Stop Hypertension (DASH) trial, that compared a typical high-fat Western diet to a low-fat, low-saturated fat (<7% of energy) diet (DASH diet) and a diet high in fruits and vegetables, showed that the low-fat diet lowered plasma LDL-cholesterol, but also lowered plasma HDL-cholesterol, compared to the typical high-fat Western diet [53]. In fact, those participants with higher baseline HDL-cholesterol saw more pronounced lowering of HDL-cholesterol from the DASH diet [53]. In another study, designed to test whether the DASH diet decreased inflammatory biomarkers for cardiovascular disease and whether underlying inflammation could impact the effects of the DASH diet, showed that the DASH diet had no effect on plasma C-reactive protein or TAG, but did lower plasma total, LDL- and HDL-cholesterol [54]. Whereas subjects with low C-reactive protein at baseline experienced greater decreases in total and LDL-cholesterol, HDL-cholesterol was reduced regardless of C-reactive protein levels at baseline [54]. TAG were increased only in those with high C-reactive protein levels at baseline [54]. The results of this study highlight the complexity of the effects of diet on plasma biomarkers for cardiovascular disease.

A Mediterranean dietary pattern has been recommended as a heart healthy diet [2]. A randomized controlled trial that studied a Mediterranean dietary pattern containing just 10% of calories from saturated fat, however, found that when compared with a control group, the Mediterranean diet had no effect on markers of inflammation or metabolic risk factors in 101 patients that had been treated for coronary artery disease [55]. Similarly, a large study conducted over 8 years in over 48,000 postmenopausal women, showed that reducing total and saturated fat intake did not reduce the risk of cardiovascular diseases and achieved only modest effects on cardiovascular risk factors [56]. Further, a multicenter, randomized, crossover-design trial that compared a typical high-fat Western diet (15% of calories from saturated fat) to a lower-saturated fat diet (9% of calories from saturated fat) and low-saturated fat diet (6% of calories from saturated fat) in over 100 men and women found that, whereas plasma total and LDL-cholesterol were decreased as saturated fat intake decreased, so was plasma HDL-cholesterol [57]. In addition, apolipoprotein (Apo) A-I, important for HDL-cholesterol function was also decreased and lipoprotein (Lp) (a) was increased [57]. This was an important finding, because Lp (a) is an often overlooked independent risk factor for cardiovascular disease. The current report of the Dietary Guidelines Advisory Committee recognizes the health benefits of following healthy eating patterns, including the Mediterranean Diet [2]. Thus, the aforementioned observations highlight the pitfalls of utilizing lipid biomarkers as sole predictors of cardiovascular risk.

### 3.2. Regular-Fat Dairy Foods: Effects on Plasma Biomarkers for Cardiovascular Disease

Few studies have evaluated regular-fat dairy foods specifically in relation to plasma biomarkers for cardiovascular disease. One study found that adding three servings of regular fat yogurt to the habitual diet decreased serum cholesterol, thus suggesting a potential hypocholesterolemic effect by yogurt consumption [58]. A study conducted in adolescent boys found that the consumption of two liters of whole milk or regular-fat yogurt per day did not lead to an increase in total or LDL-cholesterol levels as would be expected based on the saturated fat consumed, and an increase in HDL-cholesterol was higher after intake of whole milk [59]. Similarly, another study found that three weeks consumption of one liter of 2% milk, whole milk, regular-fat yogurt, skim milk, butter milk or sweet acidophilus milk did not lead to significant changes in total, LDL- and HDL-cholesterol, suggesting that in young adults large amounts of milk products were not associated with major changes in blood lipids [60]. These studies, while informative, allowed for *ad libitum* feeding and lacked detail regarding total diet, as well as detailed information regarding the inclusion criteria, physical activity and health status of participants. A randomized controlled trial, however, that tested whole vs. skim milk was since published. This study tested the diet recommended by the U.S. American Heart Association with either whole or skim milk included. Whereas total, LDL-cholesterol and ApoB levels were lower after the skim milk period compared to whole milk, there were no differences in HDL-cholesterol, non-HDL to HDL-cholesterol ratio, ApoAI and TAG between the two diets [61]. Another study that compared regular and low fat dairy indicated that low-fat dairy lowered LDL-C and IL-6 compared with high-fat dairy [62].

More research is needed to better understand the effects of regular fat dairy foods on markers of inflammation. It is important that future research recognize that cardiovascular disease is a multifactorial disease that cannot be defined by a single or narrow set of biomarkers. Reporting the effect of dairy foods on markers of biomarkers of inflammation, as well as on lipoprotein particle size and quantity may improve the capability of assessing the effects of dairy foods on cardiovascular risk.

## 4. Obesity, Cardiometabolic Risk, and Dairy Foods

Summary of lecture presented by Dariush Mozaffarian at The American Society for Nutrition Annual Meeting held in conjunction with Experimental Biology, 2015 (USA) [63]. The following text represents the authors’ summary of Dr. Mozaffarian’s lecture. Dr. Mozaffarian was not involved with the writing or publication of the text.

A systematic review of the observational literature found that although data were mixed, the majority of studies indicated regular fat dairy consumption was not associated with cardiovascular events [9]. Whereas it would be premature to assume that dairy fat is protective against cardiovascular disease, the current observational data do not support recommendations to avoid consuming regular-fat dairy foods [9]. Further, interventions focused on total and saturated fat consumption, rather than on whole foods, such as dairy foods, have proven ineffective in preventing cardiovascular events [64]. For instance, the large clinical intervention among over 5000 patients across 16 research centers, known as Look Ahead, aimed to achieve and maintain 7% weight loss in diabetic patients by focusing on reduced caloric intake and increased physical activity. It included a maximum of 30% of total calories from fat and maximum of 10% of total calories from saturated fat. The study investigators found that there was no change in cardiovascular events after the intensive lifestyle intervention [64]. The lack of effect may be because the intervention focused on fat and calorie intake, rather than on total diet quality.

A body of literature demonstrates that foods’ effects on obesity include complex influences such as on satiety, hormonal responses, hepatic de novo lipogenesis, reward and craving, gut flora responses, and metabolic expenditure that ultimately all calories are not created equal [65,66,67,68,69]. In fact, subjects on a higher-fat, low-carbohydrate diet (39% dietary fat) lost more weight and kept it off longer than subjects on a moderate fat Mediterranean diet (33% dietary fat) and a low-fat diet (30% fat) that were tested for a two-year period [70].

Weight loss can be monitored over short-term dietary interventions, but the prevention of weight gain must be monitored over the long term. This is because the average adult gains only about one pound per year. An analysis of three large cohort studies that examined diet and weight change every four years among over 100,000 subjects that participated in the Nurses’ Health Studies over 24 years indicated that certain foods were associated with weight gain and others with weight loss [71]. Cheese was found to be neutral for weight gain, as were regular, low-fat and fat-free milk [71]. Yogurt was associated with weight loss [71]. Data from the same cohort showed that high carbohydrate containing foods were associated with weight gain, and low-fat dairy foods were associated with consuming higher amounts of high carbohydrate containing foods [72]. The findings indicated that eating regular-fat foods correlated with consuming less carbohydrates and gaining less weight over time [72]. The consumption of cheese, which alone was neutral, was associated with long-term weight loss if a reduction in carbohydrate containing foods took place alongside it [72].

The evidence that regular-fat dairy foods do not contribute to weight gain is not restricted to observational data. A meta-analysis of randomized controlled trials of dairy consumption and weight change in adults indicated that total dairy consumption was associated with weight loss [73]. The same meta-analysis showed that total dairy consumption decreased fat mass and increased lean body mass [73]. In children, there have been five prospective cohort studies that have investigated milk and weight gain. Four out of the five prospective studies indicated that, whereas low-fat and fat-free milk were associated with greater weight gain, regular-fat milk was associated with less weight gain in children [74,75,76,77,78].

Obesity is an important cause of type 2 diabetes [79]. The inverse association between regular fat dairy and weight, therefore, brings into question the potential for an inverse association between regular fat dairy and type 2 diabetes. The EPIC prospective cohort, analyzed milk, cheese and yogurt separately and reported that whereas regular fat milk consumption had no association with incident diabetes, both yogurt and cheese consumption tended to be inversely associated with risk of incident diabetes [80]. In regards to dairy fat specifically, *trans*-palmitoleic acid (C16:1, *n*-7), a biomarker of dairy fat intake, was associated with 50%–60% less incidence of type 2 diabetes in an analysis from four large prospective cohorts [81]. The association between regular-fat dairy intake and type 2 diabetes warrants further investigation.

## 5. Cheese and Metabolic Diseases

Summary of lecture presented by Arne Astrup at The Federation of European Nutrition Societies 12th European Nutrition Conference, 2015 (Germany) [82].

Currently, the World Heart Federation warns consumers to beware of “bad fats”, saturated and *trans*-fatty acids, such as those present in meat and dairy foods [3]. This is despite the aforementioned body of evidence that the consumption of regular-fat dairy is not detrimental to metabolic health when compared to low-fat dairy foods [83,84]. A dose-response meta-analysis of prospective cohort studies found that dairy consumption was associated with a decreased risk of cardiovascular disease [83]. A study comparing diets high in fat from cheese, high in fat from meat, and low in fat/high in carbohydrate showed that the high fat diets raised HDL-cholesterol compared to the low-fat/high-carbohydrate diet, with no differences in LDL-cholesterol or TAG between any of the diets [85].

Despite its contribution of total and saturated fat to the diet, regular-fat dairy has not been shown to negatively impact human health (Table 1). Current evidence indicates regular-fat dairy, and in particular cheese, may actually have a beneficial effect on metabolic health. A randomized trial that tested the effect of cheese on plasma cholesterol and metabolic syndrome, as assessed by blood samples, anthropometric measurements, blood pressure and diet and lifestyle questionnaires, showed that cholesterol levels did not increase after a high intake (80 g per day) of 27% fat Gouda-style cheese [86]. In addition, participants with metabolic syndrome had reduced cholesterol at the conclusion of the intervention [86]. Another intervention study, in which Camembert cheese was fed to mildly hypercholesterolemic subjects, showed that 60 g of Camembert cheese daily did not adversely affect blood lipids or blood pressure [87]. An intervention trial showed that cheese intake in large amounts lowered LDL-cholesterol concentrations compared with butter intake of equal fat content [88]. Most recently, a systematic review and meta-analysis of randomized controlled trials investigating the effect of cheese consumption on blood lipids indicated that cheese beneficially affected plasma LDL- and HDL-cholesterol as compared to butter [89]. Although, as previously discussed, measuring HDL and LDL in isolation may not offer much insight into metabolic health. Researchers hypothesize that the unexpected effect of cheese on plasma cholesterol may be due to the cheese matrix, which includes calcium, other minerals, casein and both starter and non-starter bacterial cultures, in addition to dairy fat. Potential mechanisms may include the reduction in fat digestibility and absorption, the precipitation of calcium and fatty acids in insoluble fatty acid soaps, the precipitation of calcium and phosphate in amorphous calcium phosphate, and a potential increased fecal excretion of bile acids associated with cheese intake [90,91].

The evidence from both observational and clinical research indicates that cheese, when consumed as part of a healthy diet, is not linked to increased risk of obesity, metabolic syndrome, type 2 diabetes or cardiovascular disease. Further, cheese does not exert effects on blood lipids and blood pressure as has been traditionally predicted by its sodium and saturated fat content. Thus, the effects of cheese on metabolic health highlight the pitfalls of basing dietary guidance on nutrients, rather than foods.

## 6. Dairy Fat in Infancy

Summary of lecture presented by Bernadette Delplanque at European Federation for the Science an Technology of Lipids 12th Euro Fed Lipids Congress (France) [98].

### 6.1. Infant Formula, Breast Milk and Dairy Fat

Up until the 20th century, regular-fat cow’s milk was used to manufacture formulas to feed infants. As formulas evolved to meet specific needs of essential fatty acids, they became increasingly based on cow-milk proteins and blends of pure vegetable oils. Whereas the need for essential fatty acids are met, numerous other components of human milk, such as cholesterol fatty acids, TAG and globule fat structures are not found in pure vegetable blends. In this way, regular-fat cow’s milk is closer in fat composition to human breast milk than vegetable blends. The fatty acids and components missing from pure vegetable blends during the perinatal period may be crucial for later development and healthy adulthood [99].

Recommendations for infant formulas have been established on the premise that human breast milk is the “gold standard” that formulas should mimic. The content of lipids in human milk varies by geographical location and during lactation (3–4 g/100 g), and is mostly represented as TAG (95%). Human milk fat contains 34%–47% of saturated fatty acids (palmitic acid: 17%–25%), about 31%–43% monounsaturated fatty acids (oleic acid: 26%–36%) and about 12%–26% *n*-6 PUFA and about 0.8%–3.6% *n*-3 PUFA [99].

Linoleic acid (LA) and *alpha*-linolenic acid (ALA) are essential fatty acids present in human breast milk. ALA is a precursor for long-chain fatty acids, such as docosahexaenoic acid (DHA), which are necessary for proper brain development in infants. For decades, the ideal amount and proportion of LA and ALA has been debated, and the recommendations have changed accordingly. The LA and ALA content in breast milk depends on the mother’s diet and thus varies widely between countries; for LA, 10% to 24% of fatty acids and for ALA, 0.6% to 1.9% of fatty acids [99]. Over the last half century, the lipid composition of human breast milk changed, with higher levels of LA being observed. Ailhaud et al. [100] reported that, in France, since 1940, an increase of LA from 5% to more than 16% in 2000 has been observed. Whereas specific levels of ALA were not clearly reported, it has been established that they remained stable between the 1970s and 2000. Consequently, the LA/ALA ratio increased from about 6 to more than 16 in 2000 in certain populations, which potentially limited the bioconversion to long-chain-omega 3 fatty acids, such as DHA [101,102]. These recent modifications in human breast milk (reflecting mother’s dietary changes) are likely the largest change in infants’ diets observed in centuries considering all mammals show comparable fatty acid composition of milk [100]. Researchers speculate that this change may be linked to some metabolic diseases observed in adulthood, such as obesity and metabolic syndrome [103,104,105,106].

Recently, infant formulas based on blends of vegetable oils mimicked quite well the 20th century composition of human breast milk in terms of essential fatty acids. Not all, however, contained cholesterol, certain short- and medium-chain fatty acids or the proper sn2-position of palmitic acid on TAG. Some of these components could be provided by the addition of specific oils or products (e.g., coconut oil could contribute short- and medium-chain fatty acids).

Cow’s milk fat naturally presents fewer differences from human breast milk than vegetable blends. Cow’s milk fat offers a better representation of sn2-position of palmitic acid, has similar content of cholesterol, short- and medium-chain fatty acids (C6:0 to C12:0), all *trans*- and branched-chain fatty acids for a total of approximately 20% of milk fatty acid content. Functional purposes of the fatty acids present in cow’s milk have also been demonstrated [93]; for example, the presence of short- and medium-chain fatty acids limits the oxidation of PUFA precursors and therefore may increase bioconversion to long-chain PUFA such as DHA [107]. The presence of myristic acid is important for acylation of proteins. Furthermore, some fatty acids whose concentrations are not well established (e.g., nervonic acid) could be of importance during early brain development [108]. These fatty acids are present in breast and cow’s milk, but totally absent in vegetable formulas [99].

The most striking difference between breast milk and cow’s milk is the content of LA, which is approximately 10 times less in cow’s milk (10%–24% vs. 1.5%, respectively), while ALA is quite similar (0.7%–2% vs. 0.4%–08%). Thus, the drastic differences in the LA/ALA ratio from 10 in breast milk to less than 3 in cow’s milk could be attributed to changes in the human diet that has been observed (enriched in *n*-6) over the last 50–60 years.

Currently, all manufactured infant formulas are made of blends of vegetable oils, which contain average LA and ALA content (12%–15% and 1.5% to 2.5% respectively). The specific amounts depend upon the type and source of fats used to make the formula. The LA/ALA ratio in infant formulas varies from 5 to 15. Some formulas contain added long-chain *n*-6 and *n*-3 fatty acids (arachidonic acid (ARA) and DHA, respectively). Breast milk contains very low levels of DHA and ARA (0.1%–1% and 0.4%–0.9%, respectively) and cow’s milk contains approximately 5 to 10 times less [99].

Adequate DHA is crucial from three months before delivery to five years after birth, for rapid growth and differentiation of the brain. The addition of DHA to infant formula has not been consistently shown to have benefits in visual, neural or growth outcomes [109]. Some studies in children born preterm, however, showed that high DHA doses have been related to improvements in neurodevelopment at 18 months [110] and that a benefit could be obtained with supplementation of lactating women with DHA on psychomotor and attention of children later during childhood [111]. It is now generally recommended to include DHA in infant formulas based on vegetable oil blends to maintain a proper DHA status for infant brain and development. Addition of ARA remains controversial, however, well established procedures include the simultaneous addition of ARA when DHA is added to the formula and recent proposals recommend a lower ratio for ARA:DHA of one to 1.4 instead of the typical two [99].

### 6.2. Improvement of Long Chain-PUFA Status and Brain DHA Content with Dietary Formulas Containing Dairy Fat in an Animal Model

The effects of reintroducing dairy fat into infant formulas on blood and brain levels of DHA recently has been studied in young rats. The rat is an appropriate animal model for these nutritional studies and has been used previously to establish recommendations for infant nutrition [112,113]. Blends of different vegetable oils plus dairy fat, meeting the PUFA lipid recommendations for infant formulas (LA: 16%, ALA: 1.6%–2.5%, LA/ALA ratio: 10–5) [114,115,116,117] were compared to pure dairy fat (LA: 2.5%, ALA: 0.8%, LA/ALA ratio: 2.6) and vegetable blends with rapeseed oil rich in ALA (8%). The evaluation focused on brain levels of DHA, an important goal in neonates, since accumulation of DHA occurs during the first five years.

This study had three main findings. First, a dairy-fat-based diet (50% dairy, 50% vegetal oils, 1.5% ALA) was more efficient than a pure vegetable oil blend with the same amount of ALA (1.5%) and LA/ALA ratio (10), to increase brain DHA. Specific and complex components of dairy fat, such as the short- and medium-chain fatty acids which are highly oxidizable after absorption [118,119], may spare ALA from oxidation [120], thus favoring ALA partitioning towards desaturation and elongation pathways, increasing the long-chain *n*-3 (DHA) levels; Second, the dairy-fat-based diet (50% dairy, 50% vegetable oils) enriched with 2.3% ALA was even more efficient and this could be attributed to both the increased level of dietary ALA and the concomitant decrease in the LA/ALA ratio (from 10 to 5). A lower LA/ALA ratio has been recognized as an important factor driving the bioconversion of ALA into DHA because of the competition between the parent *n*-3 and *n*-6 fatty acids for desaturation and elongation pathways; Third, the dairy-fat-based diet containing pure dairy fat (100% dairy, 0.8% ALA, 1.9% LA) was as efficient as the rapeseed diet (100% vegetable oil, 22% LA, 8% ALA), and comparable to the 2.3% ALA dairy/vegetable blend diet, all of them containing a very low LA/ALA ratio (3 to 5). Delta6-desaturase could be involved in this process, and its role is crucial to explain these last results because ALA is the precursor of DHA but also its competitor for the last step of the delta6-desaturase pathway, and is regulated by substrate levels [121]. An excess of ALA as the first substrate could stimulate increasing quantities of some long-chain *n*-3 and secondarily limit delta6-desaturase activity in the second control point for DHA conversion, as observed for rapeseed oil, which could act as an excess precursor as shown previously [122,123]. Researchers observed a stabilization of brain DHA levels with a LA/ALA ratio of 3 to 5, suggesting there is no real need to increase the absolute amount of *n*-6 and *n*-3 precursors. Pure dairy fat with only 0.8% of ALA and a LA/ALA ratio around 3 is quite sufficient to provide required brain levels of DHA. This study also demonstrated that despite very low levels of PUFA (1.5%–3% LA, 0.5%–0.8% ALA) and a favorable LA/ALA ratio similar to rapeseed oil (maximum 3/1), pure dairy fat was able to provide adequate bioconversion of ALA to long-chain *n*-3 and DHA necessary for the brain of young animals.

Together, these observations demonstrate that brain DHA levels could be substantially improved by dairy fat based-diets. These results are similar to the results obtained in studies showing that infants fed formulas based on dairy fats [124] had better long-chain *n*-3 status than those fed formulas enriched with LA-rich vegetable oils. The use of fats that are low in PUFA such as dairy may confer some metabolic advantages in that they allow better endogenous conversion of ALA to DHA. Consequently, the use of dairy fat in infant formulas should be reconsidered, as well as the recommended absolute amount of polyunsaturated LA and ALA.

## 7. Dairy Foods, Not Nutrients: Revisiting Nutrient-Focused Guidelines

Summary of lectures presented by Dr. Dariush Mozaffarian at The American Society for Nutrition Annual Meeting held in conjunction with Experimental Biology, 2015 (USA) and Dr. Benoît Lamarche at the Dairy Nutrition Annual Symposium, 2014 (Canada) [63,125]. The following text represents the authors’ summary of lectures presented by Dr. Mozaffarian and Dr. Lamarche. Neither Dr. Mozaffarian or Dr. Lamarche was not involved with the writing or publication of the text.

The state of the science indicates that consuming lower fat dairy foods does not contribute to improved body composition and metabolic health when compared to regular fat dairy foods [83,89]. Rather, evidence-based strategies that include following overall healthy dietary patterns, of which regular-fat dairy can be a part, have been demonstrated to beneficially affect human health outcomes [71,126]. An over-emphasis on reducing nutrients to limit, as opposed to choosing healthy foods and dietary patterns, exists in dietary guidance. This focus on lowering total and saturated fat intake [126,127], can lead to policy efforts such as total calorie labeling on menus [128], and the restricted sale of regular fat dairy foods in schools [129]. The unintended consequences of such guidance could be an increased consumption of foods that are nutrient-poor and detrimentally associated with overall health [130]. Recent evidence indicates that replacing saturated fat with high-linoleic acid vegetable oils has no benefit on risk of mortality or CHD events, and may actually increase the risk of mortality from all causes [51].

Whereas more research on the relationship between dairy fat intake and cardiovascular disease and type 2 diabetes risk is warranted [9,126], the totality of current available evidence indicates that the consumption of regular fat dairy foods does not detrimentally affect human health [9,131] and is inversely associated with weight gain and the risk of obesity [9]. Dairy foods, including regular-fat milk, cheese and yogurt, can be important components of an overall healthy dietary pattern. Future research on the inclusion of regular-fat dairy foods in dietary patterns is warranted.

## Figures and Tables

**Table 1:** Summary of outcomes from systematic reviews and meta-analyses of regular-fat dairy or cheese consumption and human health outcomes.

Reference	Regular-Fat Dairy Food Included in Analysis	Outcome	Association between Regular-Fat Dairy and Outcome (Positive, Neutral, Inverse)
Ralston, 2012 [92]	Regular-fat dairy vs. low-fat dairy	Elevated blood pressure	Neutral
	Cheese vs. fluid dairy		Neutral
Soedamah-Muthu, 2012 [93]	Regular-fat dairy	Hypertension incidence	Neutral
	Cheese		Neutral
Soedamah-Muthu, 2011 [83]	Regular-fat dairy products	Cardiovascular disease (including coronary heart disease and stroke)	Neutral
		Total mortality	Neutral
Aune, 2013 [94]	Regular-fat dairy products (200 g/day)	Type 2 diabetes risk	Neutral
	Cheese (50 g/day)		Inverse
Tong, 2011 [95]	Regular-fat dairy	Type-2 diabetes	Neutral
	Whole milk		Neutral
Gao, 2013 [96]	Cheese (30 g/day)	Type-2 diabetes	Inverse
Benatar, 2013 [84]	Regular-fat dairy	Weight	Positive
		Waist circumference	Neutral
		HOMA-IR	Neutral
		Fasting glucose	Neutral
		Systolic blood pressure	Neutral
		Diastolic blood pressure	Neutral
		CRP	Neutral
Aune, 2012 [97]	Cheese (50 g/day) dose-response	Colorectal cancer risk	Neutral
